# MAP17 and SGLT1 Protein Expression Levels as Prognostic Markers for Cervical Tumor Patient Survival

**DOI:** 10.1371/journal.pone.0056169

**Published:** 2013-02-13

**Authors:** Marco Perez, Juan M. Praena-Fernandez, Blanca Felipe-Abrio, Maria A. Lopez-Garcia, Antonio Lucena-Cacace, Angel Garcia, Matilde Lleonart, Guiovanna Roncador, Juan J. Marin, Amancio Carnero

**Affiliations:** 1 Instituto de Biomedicina de Sevilla, Hospital Universitario Virgen del Rocio, Consejo Superior de Investigaciones Cientificas, Universidad de Sevilla, Sevilla, Spain; 2 Fundacion FISEVI, Instituto de Biomedicina de Sevilla/ Hospital Universitario Virgen del Rocio, Sevilla, Spain; 3 Instituto de Recherca Val d’Hebron, Barcelona, Spain; 4 Centro Nacional de Investigaciones Oncologicas, Madrid, Spain; 5 Departamento de Medicina preventiva y salud pública, Universidad de Sevilla, Sevilla, Spain; Baylor College of Medicine, United States of America

## Abstract

MAP17 is a membrane-associated protein that is overexpressed in human tumors. Because the expression of MAP17 increases reactive oxygen species (ROS) generation through SGLT1 in cancer cells, in the present work, we investigated whether MAP17 and/or SGLT1 might be markers for the activity of treatments involving oxidative stress, such as cisplatin or radiotherapy. First, we confirmed transcriptional alterations in genes involved in the oxidative stress induced by MAP17 expression in HeLa cervical tumor cells and found that Hela cells expressing MAP17 were more sensitive to therapies that induce ROS than were parental cells. Furthermore, MAP17 increased glucose uptake through SGLT receptors. We then analyzed MAP17 and SGLT1 expression levels in cervical tumors treated with cisplatin plus radiotherapy and correlated the expression levels with patient survival. MAP17 and SGLT1 were expressed in approximately 70% and 50% of cervical tumors of different types, respectively, but they were not expressed in adenoma tumors. Furthermore, there was a significant correlation between MAP17 and SGLT1 expression levels. High levels of either MAP17 or SGLT1 correlated with improved patient survival after treatment. However, the patients with high levels of both MAP17 and SGLT1 survived through the end of this study. Therefore, the combination of high MAP17 and SGLT1 levels is a marker for good prognosis in patients with cervical tumors after cisplatin plus radiotherapy treatment. These results also suggest that the use of MAP17 and SGLT1 markers may identify patients who are likely to exhibit a better response to treatments that boost oxidative stress in other cancer types.

## Introduction

MAP17 is a small (17 kDa), non-glycosylated, membrane-associated protein located in the plasma membrane and Golgi apparatus [1]. The protein sequence shows a hydrophobic amino-terminus of 13 amino acids encoding a PDZ-binding domain and two transmembrane regions [2]. MAP17 acts as an atypical anchoring site for PDZK1 and interacts with the NaPi-IIa/PDZK1 protein complex in renal proximal tubular cells [3]. The physiological role of MAP17 in proximal tubules is not well known; however, MAP17 stimulates SGLT transporters, increasing specific Na-dependent transport of mannose and glucose in *Xenopus* oocytes [1] and human tumor cells [4].

MAP17 is overexpressed, primarily through mRNA amplification, in a variety of human carcinomas [5], [6], [7]. Immunohistochemical analysis of MAP17 during cancer progression shows that overexpression strongly correlates with tumoral progression in prostate, breast and ovarian carcinomas [6], [7]. Generalized MAP17 overexpression in human carcinomas indicates that MAP17 can be a good marker for tumorigenesis and especially for malignant progression.

Tumor cells that overexpress MAP17 show increased tumoral phenotypes with enhanced proliferative capabilities in either the presence or absence of contact inhibition, decreased apoptotic sensitivity and increased migration. MAP17-expressing clones also grow more robustly in nude mice [8]. The increased malignant cell behaviors induced by MAP17 are associated with an increase in ROS production, and the treatment of MAP17-expressing cells with antioxidants results in a reduction in tumorigenic properties. The ROS increases induced by MAP17 lead to PTEN and AKT(T308)-phosphatase oxidation, maintaining AKT activation even in the absence of serum. Thus, MAP17 significantly decreases c-myc-induced caspase-3-like activity in Rat1 fibroblasts under low serum conditions, which is inhibited by ROS scavengers [9].

In contrast, Na+-dependent glucose transporter 1 (SGLT1) is the primary mediator of apical glucose uptake in tumors [10], [11]. Previous studies have demonstrated that SGLT1 activation rescues enterocytes from cell apoptosis by activating PI3K [12], and the inhibition of this membrane transport also inhibits MAP17-dependent ROS increase and proliferation [8]. Together, these results suggest that MAP17-dependent tumorigenic properties depend on the activation of ROS by SGLT1 membrane transport. SGLT1, conversely, has been previously related to cancer [13], showing a correlation with prognosis.

ROS may promote either proliferation or cell death depending on the intensity and location of the oxidative burst and the activity of the antioxidant system [14], [15]. Considering the proliferative signals delivered by ROS to cancer cells and the consequent resistance of cancer cells to pro-apoptotic signals, ROS-induced tumor cell death is more likely to be induced by ROS-generating antineoplastic therapies that increase the constitutive status above the critical threshold required for cell death.

In experimental models, ROS generation in tumors and subsequent oxidative stress are at sub-lethal levels; further ROS increases might lead tumor cells to death [7], [8], [15], [16], [17]. We hypothesized that MAP17, through SGLT1 activation, enhances the oxidative stress in tumor cells close to the threshold separating growth from death and, therefore, might be markers for tumors with high oxidative stress. Therapies increasing ROS might help cells cross this threshold and be beneficial to patients whose tumors exhibit increased levels of MAP17 and/or SGLT1.

Cervical cancer is a malignant neoplasm of the cervix uteri or the cervical area. Treatment consists of surgery (including local excision) in early stages and chemotherapy plus radiotherapy in advanced stages of the disease. Current standard treatment includes radiotherapy and brachytherapy plus cisplatin, which are ROS-inducing therapies, depending on the stage of the tumor [18], [19].

Patient prognosis depends on the cancer stage. The overall 5-year survival rate is approximately 72% [19], [20]. With treatment, 80 to 90% of women with stage I cancer and 50 to 65% of those with stage II cancer survive to 5 years after diagnosis. However, only 25 to 35% of women with stage III cancer and 15% or fewer of those with stage IV cancer are alive after 5 years [19], [20].

In the present work, we explored whether increases in MAP17 and its effector SGLT1 serve as prognostic markers for improved survival in patients with cervical tumors currently treated with cisplatin and radiation therapy.

## Materials and Methods

### Patient Selection

This research followed the tenets of the Declaration of Helsinki and was approved by the Hospital Val d’Hebron institutional ethical review board. All samples were obtained after informed consent was provided by the patients. The participants provided their written informed consent to participate in the study. The patients included in this retrospective study were selected from a clinical database at Hospital Val d’Hebron.

### Tissue Acquirement and Preparation

Human cervical carcinoma tissues were obtained from surgical procedures and sent to the pathology laboratory for diagnosis. The tissues were diagnosed using routine hematoxylin and eosin stain. Tumor and normal counterparts from the remaining specimen were saved in a tumor bank for subsequent experiments.

### Immunohistochemistry

Paraffin-embedded tumoral samples were kindly provided by the Pathology Department at Hospital Val d’Hebron. Three-micrometer slices were sectioned from the TMA block and applied to coated, immunochemistry slides (DAKO, Glostrup, Denmark). The slides were baked overnight in a 56°C oven, deparaffinized in xylene for 20 min, rehydrated through a graded ethanol series and washed with PBS. A heat-induced epitope retrieval step was performed by heating a slide in a solution of sodium citrate buffer pH 6.5 for 2 min in a conventional pressure cooker. After heating, the slides were incubated with proteinase K for 10 min and rinsed in cool running water for 5 min. Endogenous peroxide activity was quenched with 1.5% hydrogen peroxide (DAKO) in methanol for 10 minutes, and incubation with the primary antibodies anti-MAP17 (1∶4) and anti-SGLT1 (Abcam #14685) was performed for 40 min. After incubation, immunodetection was performed with the EnVision (DAKO, Glostrup, Denmark) visualization system using diaminobenzidine chromogen as the substrate, according to the manufacturer’s instructions. Immunostaining was performed in a TechMate 500 automatic immunostaining device (DAKO) and measured through a double-blind visual assessment using microscopic observation according to the anatomopathological experience of pathologists.

A monoclonal MAP17 antibody was generated from bacterial-purified GST-MAP17 protein. Several clonal antibodies were tested for specificity and validated through antigen competition (See [21] for full characterization).

### Immunodetection of MAP17

The cells were trypsinized and cytospin onto glass coverslips. The following day, the cells were fixed with acetone for 10 min and then incubated with MAP17 monoclonal antibody for 30 min. The cells were washed three times with PBS and incubated for additional 30 min with a secondary goat anti-mouse antibody (DAKO Cytomation) diluted 1∶50 in fetal bovine serum. After incubation, immunodetection was performed with the EnVision (DAKO, Glostrup, Denmark) visualization system using diaminobenzidine chromogen as the substrate. After washing, the slides were mounted with Aquatex (Merck). The images were acquired using an Olympus Provis Microscope AX70.

### Cell Culture

Hela malignant cervical tumor cells were obtained from the ECACC human cell line repository and maintained in Dulbeccós modified Eaglés medium (Sigma) containing 10% fetal bovine serum (Sigma), penicillin, streptomycin and fungizone. MAP17 full-length cDNA was cloned into pBabe puro and mass culture generated by stable gene transfer in Hela cells. After selection with 2 µg/ML puromycin, mass cultures were used for the study. As a control, Hela cells were transfected with pBabe puro alone and selected.

### Cytotoxicity Studies

Cytotoxicity studies were performed as described previously [22]–[23]. Briefly, 4,000 cells/well were seeded in 96-well plates and left to attach and grow for 24 hours before treatment. The drugs were weighed and then diluted with DMSO to generate a 10-mM solution. From here, a “mother plate” with serial dilutions was prepared at 200X the final concentration in culture. Eleven serial dilutions of the drug from an initial 30-µM dose were assayed per compound in a minimum of three independent experiments. The medium was removed from the cells and replaced with 0.2 ml of medium dosed with drug. Each concentration was assayed in triplicate. Two sets of control wells were included on each plate consisting of either medium only or medium with the same concentration of DMSO. A third control set was established consisting of untreated cells just before addition of the drugs (as the seeding control; the number of cells starting the culture). The cells were exposed to drug for 96 hours and then incubated with 3-(4,5-dimethylthiazol-2-yl)-2,5-diphenyltetrazoliumbromide (MTT) substrate. The resulting absorbance was measured by means of a microplate reader at 410 nM (Bio-Rad, Hercules, CA, USA), and the cytotoxic effect of each treatment was assessed by calculating the concentration necessary to induce 50% cell death (the IC50 value) using PRISM software.

### Analysis of the Transcription of Genes Regulating ROS

Total RNA was purified using the TRI-REAGENT (Molecular Research Center, Cincinnati, OH, USA). Reverse transcription was performed with 5 µg of mRNA using MMLV reverse transcriptase (Promega) and oligo dT primer, according to the manufacturer’s recommendations. RNA was collected from all samples, and reverse transcription was performed using the RT2 First Strand kit (SuperArray Biosciences, Frederick, MD, USA). Polymerase chain reactions (PCR) were performed to evaluate expression of 84 genes using the RT2 profiler PCR array (Human Oxidative Stress and Antioxidant Defense SuperArray) on the ABI Fast 7900 with RT2 Real-Time SYBR green PCR master mix PA-011. The thermocycler parameters were 95°C for 10 min followed by 40 cycles of 95°C for 15 s and 60°C for 1 min. Relative changes in gene expression levels were calculated using the comparative threshold cycle (ΔΔCt) method. This method first subtracts the Ct (threshold cycle number) of the gene-average Ct of the three housekeeping genes on the array (HPRT1, GAPDH, and ACTB) to normalize the RNA amount. Finally the ΔΔCt was calculated as the normalized average Ct of the test group, vs the normalized average Ct of the houakeeping genes group. This ΔΔCt value was raised to the power of 2 to calculate the degree of change. (http://www.sabiosciences.com/rt_pcr_product/HTML/PAHS-065Z.html).

### Quantitative RT-PCR

To measure human MAP17 expression, real-time PCR was performed using the ABI 7900HT cycler (Applied Biosystems). The reaction was performed in 96-well plates, and QPCR reactions were run using Taqman Gene Expression assays (Applied Biosystems). The detection of b-actin was used as an internal control. Relative quantification values were expressed as log10 of relative quantity. Relative quantity and statistical analyses for QPCR data were calculated using Applied Biosystem RQ Manager 1.2.1 software.

### Western blot Analyses

Western blot analyses were performed as described previously [24], [25]. Briefly, the cells were washed twice with PBS and lysed by sonication in lysis buffer (50 mM Tris-HCl, pH 7.5; 1% NP-40; 2 mM Na3 VO4; 150 mM NaCl; 20 mM Na4P2O7; 100 mM NaF; and complete protease inhibitor cocktail; Roche Molecular Biochemicals). The samples were separated on 7.5–15% SDS-PAGE gels, transferred to PVDF membranes (Immobilon-P, Millipore) and immunostained. The following primary antibodies were used: MAb anti-SGLT1 αSGLT1 (Abcam #14685); and MAb anti-α-tubulin (Sigma 9026). Horseradish peroxidase-labeled rabbit anti-mouse (Promega diluted 1∶5000) and goat anti-rabbit (Calbiochem 401315, diluted 1∶4000) secondary antibodies were used, respectively. The proteins were visualized using the ECL detection system (Amersham Biosciences).

### Glucose Transport Assays

A total of 10^5^ logarithmically growing cells were seeded in 24-well plates and preincubated with 1 µM glucose (Sigma Chemical Co, St. Louis, MO, USA) for 1 h at 37°C. Then, 50 µCi/ml of D-[2–3H] glucose (Amersham Biosciences, Buckinghamshire, UK) was added and incubated for 4 h at 37°C. After washing to remove the unincorporated radioactivity, the cells were lysed (50 mM Tris-HCl pH 7.5, 1% NP-40, 150 mM NaCl) and prepared for counting with an aqueous scintillation cocktail in Wallac 1414.

## Results

### MAP17 Overexpression in Hela Human Cervical Tumor Cells Alters the RNA Expression Levels of Genes Regulating ROS

**Figure 1:**
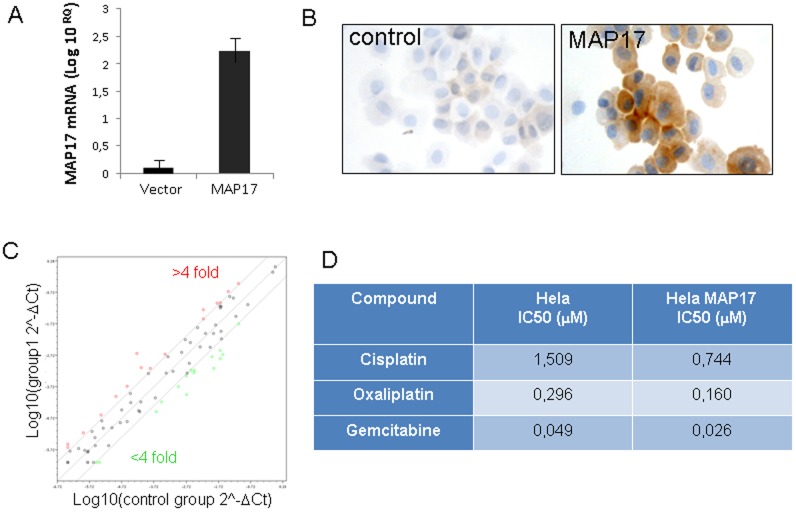
MAP17 overexpression in Hela cells. Hela cancer cells expressing ectopic MAP17 cDNA were selected and analyzed for MAP17 protein expression by A) quantitative measurement of MAP17 mRNA expressed ectopically in Hela cells and B) immunodetection after cytospin centrifugation onto slides. C) Map17 alters the transcription of genes involved in oxidative stress. A graph is shown depicting the distribution of gene transcriptional alterations induced by MAP17 in Hela cells. D) The IC50 values are shown for different chemotherapeutic drugs in Hela cells expressing MAP17 or with vector only.

To first explore whether cervical cells alter ROS regulation by expressing MAP17, we ectopically expressed MAP17 cDNA into Hela cervical tumor cells (Figures 1A and 1B). After selection, we analyzed the transcription of 84 genes regulating ROS (RT^2^ Profiler™ PCR Array from Sabioscience). We found that in approximately 30% of genes, the RNA levels varied more than 4-fold (Figure 1C). Some genes, such as PTGS1 (39-fold increase) or SIRT2 (10-fold increase), were clearly upregulated, while BNIP3 or MSRA (18-fold decrease) were downregulated (Table 1). These data clearly indicate that MAP17 overexpression in Hela cells altered the RNA levels of genes regulating oxidative stress, and this altered the levels of ROS produced in Hela cells (Figure S1). Next, we sought to determine whether MAP17 also altered the response to chemotherapeutic drugs known to activate oxidative stress. Hela cells carrying MAP17 cDNA or empty vector were subjected to standard cytotoxic assays [26], [27] with 11 different concentrations of cisplatin, oxaliplatin or gemcitabine. We found that the cells with MAP17 were more sensitive (more than 2-fold) than control cells (Figure 1D and Figure S2). Therefore, our data indicate that MAP17 altered the transcriptional balance of genes involved in ROS metabolism in cervical tumor cells, and this imbalance may increase cell sensitivity to chemotherapeutic drugs that induce ROS.

**Table 1:** Genes involved in ROS regulation in which transcription is significantly altered by MAP17 expression in Hela cells.

Genes upregulated >4-fold		Genes downregulated >4-fold	
Gene Symbol	Fold Change	Gene Symbol	Fold Change
CCL5	7,3958	ALB	−4,3486
GPX3	6,9993	ATOX1	−7,0806
KRT1	5,5198	BNIP3	−18,7871
MGST3	5,1573	CAT	−6,293
PRDX2	8,0437	DHCR24	−5,3102
PRDX5	4,054	DUOX1	−5,2595
PRG3	7,0221	GPX1	−4,3298
PTGS1	39,1571	MSRA	−18,4663
PXDNL	5,2399	NCF2	−7,5352
SCARA3	4,9336	OXR1	−4,2456
SIRT2	9,8365	OXSR1	−5,6898
SOD2	4,1722	PRDX3	−8,1426
SOD3	5,5476	PRDX4	−13,334
SRXN1	4,9745	SELS	−4,7526
TXNRD1	4,4648	STK25	−5,165
TXNRD2	5,0278	HPRT1	−14,7514

### MAP17 Overexpression in Human Cervical Tumors Correlates with Advanced Stage Disease

MAP17 is overexpressed in carcinomas, specifically in ovarian, prostate and breast carcinomas, and its overexpression correlates with advanced stages [6]. Therefore, we wanted to confirm whether MAP17 expression is a marker for human cervical tumorigenesis. To assess MAP17 protein expression in these tumors, we analyzed 239 cervical tumor samples using immunohistochemistry. In all cases, the sample was obtained from a biopsy prior to treatment, and in all cases, treatment was homogeneous (Table 2), consisting of radio plus brachytherapy combined with 6 cycles of cisplatin. Most tumors were TNM stages IB, IIA and IIB; however, there were several tumor types represented. The different tumors included 97 squamous carcinomas, 32 mucinous carcinomas, 12 endometroid carcinomas, 10 clear cell carcinomas, 8 serous type, 7 transitional type, 9 undifferentiated, 7 adenosquamous carcinomas, and 3 adenomas. Furthermore, the cohort contained 54 tumors of an unidentified type.

**Table 2:** The characteristics of the patients included in the study.

Sex		Female 100%	
**Age**		26–97	Average 55,67
**Grade**	1	13,6%	(n = 36)
	2	36,3%	(n = 96)
	3	32,2%	(n = 85)
	Nd	17,8%	(n = 47)
**Stage**	IA	1%	
	IB	60%	
	IIA	20%	
	IIB	18%	
	III	1%	
**Treatment**	Radiotherapy (50 Gy) + brachytherapy (15 Gy) + cisplatin (6 cycles: 40 mg/m2)		

**Figure 2:**
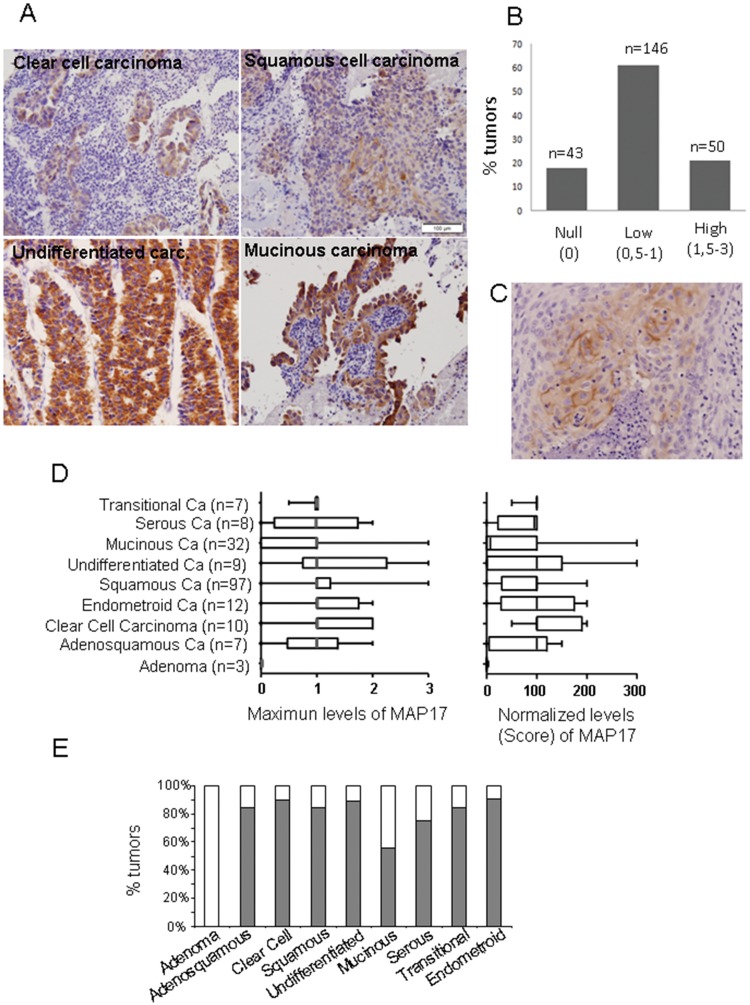
MAP17 overexpression in cervical tumors. A) Representative images of MAP17 immunostaining are shown for different tumor types. B) A graph is shown representing the percentage of cervical tumors with different MAP17 levels. C) Immunostaining was performed showing membrane-only distribution of MAP17. D) The distribution of the MAP17 expression levels among different cervical tumor types is shown. The maximum-levels graph refers to the maximum staining intensity observed in any part of the tumor (scale from 0 to 3). The normalized-levels (score) graph refers to maximum levels (0–3) scored by the percentage of cells (0–100). The normalized levels were obtained by multiplying the percentage of cells by the level of intensity observed. E) A graph representing the percentage of MAP17-positive tumors by tumor subtype is shown.

The analysis of MAP17 levels in the set of tumors showed that only 18% of all tumors were negative for MAP17 staining, while the remaining 82% were positive to differing degrees. Figure 2A shows representative images of MAP17 staining of some cervical tumor types.

The staining results were evaluated at the microscopic level according to visual criteria and were designated as the following: 0 = no staining; 0,5 = very weak staining; 1 = positive staining, clear but weak; 1,5 = positive staining; 2 = positive, strong staining; and 3 = positive, very strong staining. We also annotated the percentage of tumoral cells positive for MAP17 to score the overall levels of MAP17 observed. Out of the 239 tumors, only 43 (18%) did not express MAP17. A total of 146 (61%) showed intermediate levels, and 50 tumors (21%) showed very high MAP17 expression levels (Figure 2B).

We observed two different subcellular distributions of MAP17. The first distribution pattern involved cytoplasmic and membranous staining, with broad cytoplasmic distribution, especially in the perinuclear region (Figure 2A). The second distribution pattern showed MAP17 staining only in the membrane (Figure 2C). Most MAP17-positive tumors showed a cytoplasmic plus membranal distribution; however, approximately 5% of samples showed a clear membrane-only distribution. Remarkably, a few tumors contained different populations showing both subcellular localizations for MAP17; we do not currently know the reason for such differential distributions. In cultured cells, MAP17 shows primarily perinuclear cytoplasmic staining with some plasma membrane localization. It is possible that the strong anchoring of MAP17 to intracellular membranous compartments, such as the Golgi, accounts for this intracellular localization. Therefore, it is also possible that some tumors accumulate intracellular membranous compartments and accumulate increased levels of MAP17 in the cytosol, while in other tumors, the level of internal membranes is minimal, and the cells accumulate MAP17 in the cellular membrane. It may also depend on the molecular context of the specific tumor or cellular clone. However, more research is required to understand this distribution variability.

Next, we analyzed the MAP17 distribution levels for different tumor types. For this task, we evaluated MAP17 levels in two different ways: we determined the maximum level of MAP17 found in the tumor; or we normalized the levels by multiplying the MAP17 signal intensity by the percentage of positive cells to calculate the score (Figure 2D). We found that most tumor types showed a broad range of levels with a median of 1: low but clear levels of MAP17. However, mucinous type tumors appeared to express very low MAP17 levels. Remarkably, adenoma benign tumors did not express MAP17. Figure 2E shows the percentage of each tumor type that showed positivity (at any level) for MAP17.

### MAP17 Overexpression in Hela Tumor Cells Increases Glucose uptake and SGLT1 Content

**Figure 3:**
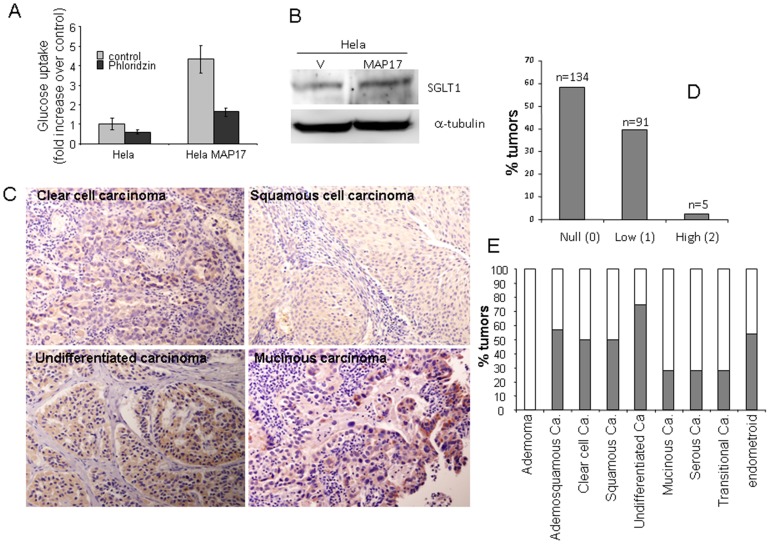
SGLT1 overexpression in cervical tumors. A) MAP17 expression in Hela cells increases glucose uptake that is blocked by the SGLT inhibitor phloridzine. B) Western blot analysis was performed for SGLT1 in Hela cells expressing MAP17 or vector alone (V). C) Representative images are shown of SGLT1 immunostaining of different cervical tumor subtypes. D) A graph is shown representing the percentage of cervical tumors with different SGLT1 levels. E) A graph is shown representing the percentage of SGLT1-positive tumors by tumor subtype.

Previous results have demonstrated that SGLT1 activation rescues enterocytes from cell apoptosis by activating PI3K [12], and the inhibition of this membrane transport inhibits MAP17-dependent ROS increases and proliferation [8]. To explore the relationship of this gene with MAP17 in the cervix, we first measured glucose uptake in Hela cells expressing MAP17 (Figure 3A). It was found that glucose uptake was increased an average of 4-fold and was inhibited upon treatment with the SGLT inhibitor ploridzine. Furthermore, we found that SGLT1 protein levels increased an average of 2-fold in Hela cells expressing MAP17 (Figure 3B).

### SGLT1 Overexpression in Human Cervical Tumors Correlates with MAP17 Levels

These and other, previous results indicate that MAP17-dependent tumorigenic properties depend on the indirect activation of ROS by SGLT1 transport. Therefore, we measured SGLT1 expression levels in the same cohort of cervical tumor samples.

We found that the tumors showed positive SLGT1 staining (Figure 3C), with approximately 40% tumors being positive for SLGT1 (Figure 3D). However, only a few samples showed very high staining levels. The distribution of the SLGT1-positive tumors among the different cervical tumor types showed a pattern similar to MAP17; adenoma benign tumors were clearly SLGT1 negative (Figure 3E).

**Figure 4:**
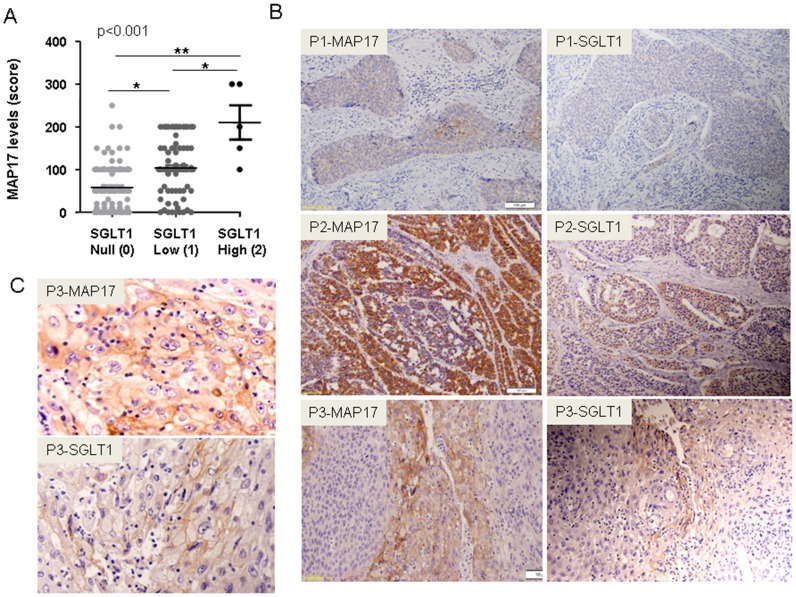
The correlation between MAP17 and SGLT1 expression levels in the same tumor samples. A) A graph is shown depicting MAP17-SGLT1 correlations in all samples analyzed. Statistical analyses were performed using a 1-way ANOVA. B) Three representative examples of a positive correlation are shown (P1, P2 and P3 are 3 different tumor samples). Two samples of each tumor were stained for MAP17 and SGLT1. C) Magnification is shown of the P3 figures shown in B. Images show the plasma membrane distribution of MAP17 and SGLT1 in the sample tissue sample.

Given the functional relationship between MAP17 and SLGT1, we analyzed whether a correlation between the expression levels of both proteins existed. We grouped the SGLT1 expression levels as low (0–0,5), medium (0,5<x<1,5) or high (>1,5), and compared the groups according to MAP17 expression levels (Anova, Krustell-Wallis test). We found a direct, significant correlation between MAP17 and SGLT1 levels within the groups (p<0,001; Figure 4A). Next, we compared the levels of both proteins in the same tumor samples and found a significant correlation (Spearman test, p<0,001) between the levels of both proteins in the same tumor sample.

Again, we observed 2 different subcellular distributions for SGLT1: cytoplasmic and membranous and membrane only. Clearly, SGLT1 distribution patterns matched MAP17 distribution patterns in the same tumor (Figure 4B).

### MAP17 and SGLT1 Expression Levels Correlate with Survival in Patients with Cervical Tumors

Because the expression of MAP17 increases ROS [8], which might be acting as a second messenger to increase the tumorigenic properties of cancer cells, we hypothesized that further increases in ROS might increase ROS levels beyond threshold and turn the physiology of the cells towards apoptosis. We observed (Figure 1D) that Hela cells overexpressing MAP17 increased by 2-fold their sensitivity to several chemotherapeutic drugs known to induce ROS. Therefore, MAP17 might be a marker for the activity of treatments where oxidative stress plays a key role in the response, such as cisplatin or radiotherapy.

**Figure 5:**
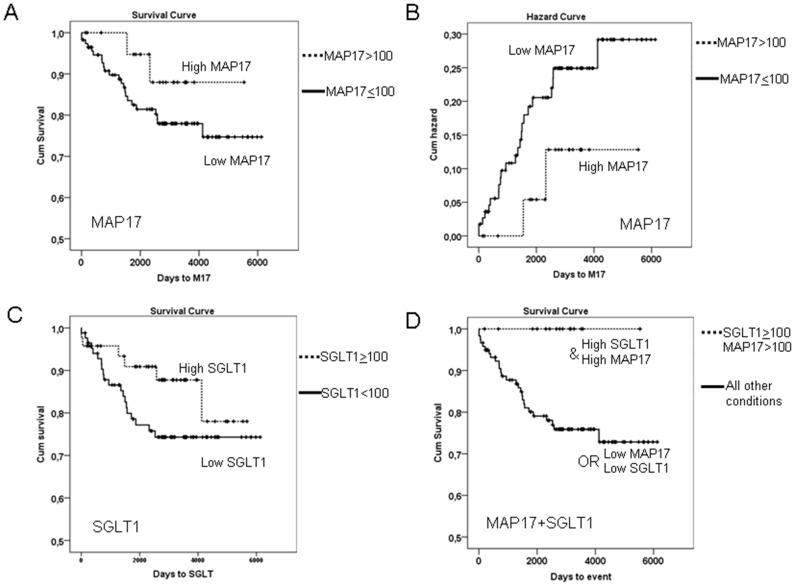
MAP17 and SGLT1 levels and the combination are good independent markers for survival. A) A Kaplan-Meier curve is shown indicating that MAP17 could be a good prognostic marker for survival in cervical tumor patients treated with cisplatin and radiotherapy. B) The impact function indicates the accumulated impact of having high MAP17 levels in the survival of cervical tumor patients treated with cisplatin and radiotherapy. C) A Kaplan-Meier curve is shown indicating that SGLT1 levels could be a good prognostic marker for survival in cervical tumor patients treated with cisplatin and radiotherapy. D) A Kaplan-Meier curve is shown indicating that high levels of MAP17 and SGLT1 levels are good prognostic markers for survival in cervical tumor patients treated with cisplatin and radiotherapy.

After the samples were collected, the patients of our cohort were treated with standard treatment for this type of tumor, radiotherapy (50 Gy) + brachytherapy (15 Gy) + cisplatin (6 cycles: 40 mg/m2). We analyzed whether MAP17 levels influenced response to therapy. To that end, we explored whether the different MAP17 levels correlated with patient survival. Kaplan-Meier curves showed that patients with tumors with medium or high levels of MAP17 expression levels (>100 normalized score) showed improved survival than patients with tumors with low or null levels of MAP17 (Figure 5A). Overexpression of MAP17 had a clear impact on patient survival (Figure 5B).

Therefore, a high level of MAP17 correlated with improved survival and is a good prognostic factor in cervical cancer treated with radiotherapy and cisplatin.

On the other hand, previous studies have demonstrated that inhibition of SGLT1 membrane transport also inhibit MAP17-dependent ROS increases and proliferation [5]. Because the expression of SGLT1 correlated with MAP17, we studied whether the presence of SGLT1 in the same cohort of tumors was related to prognosis independently and in connection with MAP17.

Similar to MAP17, SGLT1 levels showed a clear correlation with patient survival. The patients whose tumors expressed medium or high levels of SGLT1 (normalized levels ≥100) showed improved survival than patients with low SGLT1 levels (Figure 5C). However, the combination of both markers showed a clear impact on patient survival. When exhibiting a combination of high MAP17 levels (>100) and high SGLT1 levels (≥100), patients showed complete survival (p<0.001; Figure 5D), while the patients with low MAP17 or low SGLT1 levels, or both, had the worst prognosis (Figure 5D).

## Discussion

ROS are beneficially involved in many signaling pathways that control development and maintain cellular homeostasis [15]. Under physiological conditions, a tightly regulated redox balance protects cells from injurious ROS activity. However, if altered, ROS promote various pathological conditions including cancer [16], [28], [29], [30], [31], [32], [33]. Understanding the duality of ROS as cytotoxic molecules and key mediators in signaling cascades may provide novel opportunities to improve cancer therapeutic interventions.

MAP17 is overexpressed, primarily through increased mRNA levels, in a variety of tumors. Because MAP17 expression increases ROS through SGLT1 in cancer cells, we hypothesized that MAP17 and SGLT1 might be markers for tumors with high oxidative stress, and therefore, a further increase in ROS might elevate the levels beyond the apoptotic threshold. In addition, MAP17 and/or SGLT1 might be markers for the activity of treatments where oxidative stress plays a key role in the response, such as cisplatin or radiotherapy.

MAP17 and SGLT1 were clearly expressed in a percentage of cervical tumors of different types. Furthermore, there was a significant correlation between the expression of MAP17 and SGLT1 in the same sample. High levels of either MAP17 or SGLT1 correlated with improved patient survival after treatment. In addition, all patients who showed combined high MAP17 and SGLT1 expression were alive at the end of this study. Therefore, combined high MAP17 and SGLT1 expression is a marker for good prognosis in cervical tumors after cisplatin plus radiotherapy treatment.

MAP17 protein is overexpressed in a large percentage of the tumors analyzed and significantly correlated with the tumor grade in ovarian, prostate and breast carcinomas [6], [7]. In tumors such as breast, ovary, colon, stomach, cervix and thyroid gland, the percentage of overexpression in tumor samples is higher than 70%, while in lung, uterus and rectum, it is approximately 50%. These results suggest that MAP17 and SGLT1 markers might be used to identify patients likely to exhibit a better response to treatments boosting oxidative stress in other cancer types. In fact, cervical carcinoma cells overexpressing MAP17 are more sensitive to agents capable of inducing oxidative stress.

The increased tumorigenic properties induced by MAP17 are associated with an increase in ROS because MAP17 greatly alters the mRNA levels of genes involved in oxidative stress and increases endogenous ROS, and the antioxidant treatment of MAP17-expressing cells entails a reduction in the tumorigenic properties of these cells [8], [9]. Therefore, ROS is generated in a MAP17-dependent manner as an intracellular signal and induces a growth-related genetic program. On the other hand, Na-dependent glucose transporter 1 (SGLT1) appears to mediate MAP17-induced intracellular glucose uptake and ROS increases [8]. Together, these results suggest that MAP17-dependent tumorigenic properties depend upon the activation of ROS through glucose increases by SGLT1 membrane transport.

A low level of ROS is indispensable for several physiologic cell processes including proliferation, apoptosis and cell death [34]. A mild increase in ROS has been shown to activate signaling cascades that can strongly influence the regulation of cell growth and tumorigenic processes ([7], [28], [29], [30], [31], [32], [33], [35], [36]. However, a further increase in ROS levels raises oxidative stress and creates a potentially toxic cellular environment. Under normal physiological conditions, a balance between ROS generation and oxidative defenses exists in the cell. In these defenses, endogenous antioxidant enzymes play a significant role. Enzymes such as superoxide dismutase (SOD) and catalase (CAT), which act on O2- and H2O2, respectively, glutaredoxins, glutathione peroxidases (GPXs), which use glutathione as a co-substrate, peroxiredoxins and thioredoxins are in a delicate balance with oxidative inputs [35]. Although many cells can tolerate limited doses of ROS, when the balance tips further in favor of ROS, programmed cell death is initiated [37]. Excessive ROS accumulates that cellular detox enzymes cannot neutralize in the chemical cellular environment, especially within the mitochondria, initiating the cell death program.

Therefore, we can hypothesize that tumors, not just cervical, expressing high levels of ROS producing MAP17 and SGLT1 proteins can benefit from therapies that increase oxidative stress, not only cisplatin and radiotherapy, even if they are not the first therapeutic option. Various chemotherapeutic agents including platinum derivatives, doxorubicin or camptothecin have redox-mediated activity on tumor cells [38], [39] without effects on healthy tissues [40]. Furthermore, patients could also benefit from combined therapies in which, along with the cytotoxic chemotherapy that induces ROS (i.e., cisplatin and doxorubicin), a boost of the oxidative stress is induced by a specific pro-oxidative agent (not necessarily the antitumor drug). These combinations of ROS-inducing chemotherapy plus pro-oxidant therapies could result in good antitumor activity. The first attempt to employ pro-oxidant agents in vivo was reported by Nathan and Cohn in 1981 using glucose oxidase as an H2O2 precursor, obtaining a significant decrease in tumor growth [41].

Because cells can develop adaptive responses to ROS, mostly through increases in detoxifying enzymes [42], we can hypothesize that the inhibition of classic oxidative stress detoxification enzymes may increase the efficacy of certain antitumor therapies that increase ROS [43], at least in tumors overexpressing MAP17 and/or SGLT1. However, the delicate balance between oxidative stress, cancer and cell death make new experimental tests necessary to acquire a deep understanding of these processes.

## Supporting Information

Figure S1 **MAP17 expression increases ROS in Hela cells.** To visualize intracellular ROS levels, cells grown on coverslips were washed twice with warm PBS and then incubated at 37°C with 8 µM of CM-H2DCFDA (Molecular Probes) in warm PBS supplemented with 2.5 mM glucose for 15 min. Then, PBS was replaced with 10% FBS supplemented DMEM, and cells were incubated 10 min in the same conditions. Cells were washed with warm PBS and fixed with 4% paraformaldehyde (Sigma) at room temperature for 5 min. The fixed cells were washed three times with PBS and the coverslips mounted in mowiol. Intracellular ROS were visualized using a Confocal Ultra-spectral microscope Leica TCS-SP2-AOBS-UV. Percentage of cells showing immunofluorescence was calculated and refered to parental cells (100%). The experiment was repeated 3 times independently.
(DOC)

Figure S2 **Cytotoxicity curves for different drugs.**
(DOC)
