# Supplementary figures and images for: MAP17 and SGLT1 Protein Expression Levels as Prognostic Markers for Cervical Tumor Patient Survival
Source: PLoS One. 2013 Feb 13;8(2):e56169. doi: 10.1371/journal.pone.0056169 (PMC3572015; doi:10.1371/journal.pone.0056169)

**Supplementary figure 1: MAP17 expression increases ROS in Hela cells**

**
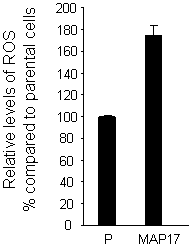
**

Supplement: Figure S1 — MAP17 expression increases ROS in Hela cells. To visualize intracellular ROS levels, cells grown on coverslips were washed twice with warm PBS and then incubated at 37°C with 8 µM of CM-H2DCFDA (Molecular Probes) in warm PBS supplemented with 2.5 mM glucose for 15 min. Then, PBS was replaced with 10% FBS supplemented DMEM, and cells were incubated 10 min in the same conditions. Cells were washed with warm PBS and fixed with 4% paraformaldehyde (Sigma) at room temperature for 5 min. The fixed cells were washed three times with PBS and the coverslips mounted in mowiol. Intracellular ROS were visualized using a Confocal Ultra-spectral microscope Leica TCS-SP2-AOBS-UV. Percentage of cells showing immunofluorescence was calculated and refered to parental cells (100%). The experiment was repeated 3 times independently. (DOC) [file pone.0056169.s001.doc]

**Supplementary figure 2: Cytotoxicity curves for different drugs.**


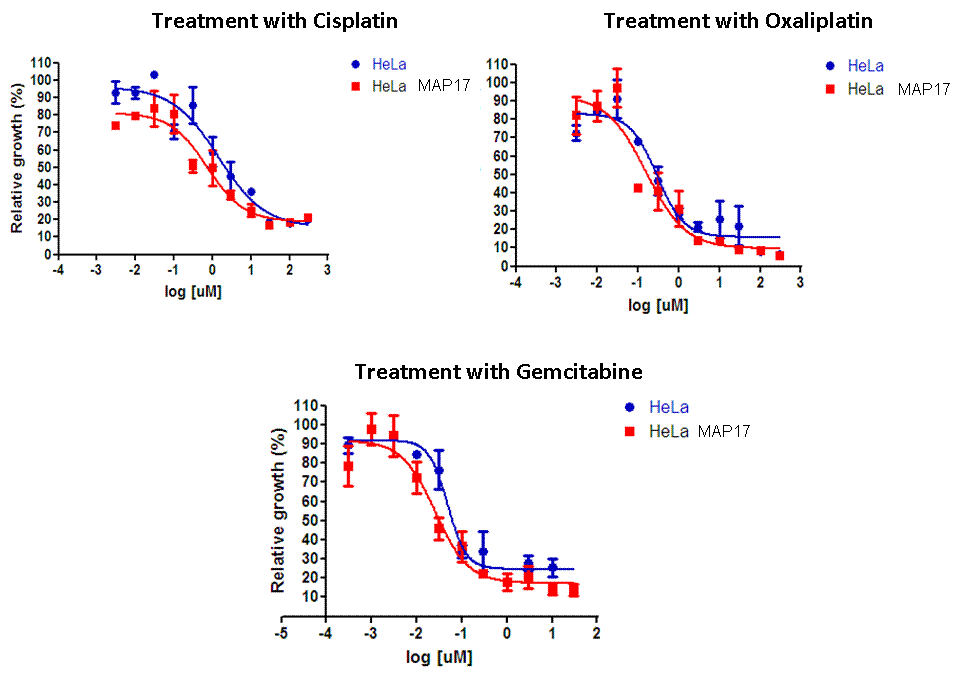

Supplement: Figure S2 — Cytotoxicity curves for different drugs. (DOC) [file pone.0056169.s002.doc]
